# Supplementary material for: Biochar Suppresses Bacterial Wilt of Tomato by Improving Soil Chemical Properties and Shifting Soil Microbial Community
Source: Microorganisms. 2019 Dec 10;7(12):676. doi: 10.3390/microorganisms7120676 (PMC6955753; doi:10.3390/microorganisms7120676)
Supplement: Supplementary file 1 [file microorganisms-07-00676-s001.zip › Supplementary files /Table S4.docx]

**Table S4**. Correlations between soil chemical properties and soil bacterial community at the phylum level.

|  | **pH** | **TOC** | **TN** | **SOM** | **Available N** | **Available P** | **Available K** | **Urease** | **Acid phosphatase** | **Catalase** | **Acidobacteria** | **Bacteroidetes** | **Gemmatimonadetes** | **Proteobacteria** | **Nitrospira** |  |
| --- | --- | --- | --- | --- | --- | --- | --- | --- | --- | --- | --- | --- | --- | --- | --- | --- |
|  |  |  |  |  |  |  |  |  |  |  |  |  |  |  |  |  |
| **pH** | 1 | .705* | .679* | ns | .597* | .836** | .871** | ns | .673* | ns | -.726** | .717** | .648* | ns | ns |  |
| **TOC** |  | 1 | .956** | ns | ns | .774** | .905** | ns | .695* | ns | -.621* | ns | ns | ns | ns |  |
| **TN** |  |  | 1 | ns | ns | .671* | .814** | ns | ns | ns | ns | ns | ns | ns | ns |  |
| **SOM** |  |  |  | 1 | ns | ns | ns | ns | ns | ns | ns | ns | ns | ns | ns |  |
| **Available N** |  |  |  |  | 1 | 0.564 | .606* | ns | ns | ns | -.577* | .630* | ns | ns | ns |  |
| **Available P** |  |  |  |  |  | 1 | .934** | .649* | .830** | ns | -.764** | .685* | .701* | ns | ns |  |
| **Available K** |  |  |  |  |  |  | 1 | .581* | .823** | ns | -.792** | .717** | .679* | ns | ns |  |
| **Urease** |  |  |  |  |  |  |  | 1 | ns | ns | -.628* | .740** | ns | .723** | .667* |  |
| **Acid phosphatase** |  |  |  |  |  |  |  |  | 1 | ns | -.808** | .658* | ns | ns | ns |  |
| **Catalase** |  |  |  |  |  |  |  |  |  | 1 | ns | ns | ns | ns | ns |  |
| **Acidobacteria** |  |  |  |  |  |  |  |  |  |  | 1 | -.878** | -.663* | -.632* | ns |  |
| **Bacteroidetes** |  |  |  |  |  |  |  |  |  |  |  | 1 | .617* | .833** | ns |  |
| **Gemmatimonadetes** |  |  |  |  |  |  |  |  |  |  |  |  | 1 | .614* | .607* |  |
| **Proteobacteria** |  |  |  |  |  |  |  |  |  |  |  |  |  | 1 | .738** |  |
| **Nitrospira** |  |  |  |  |  |  |  |  |  |  |  |  |  |  | 1 |  |

(TOC: Total organic carbon; TN: Total nitrogen; SOM: Organic matter; *p*<0.05,*; *p*<0.01,* *, Pearson text)
