# Supplementary material for: Biochar Suppresses Bacterial Wilt of Tomato by Improving Soil Chemical Properties and Shifting Soil Microbial Community
Source: Microorganisms. 2019 Dec 10;7(12):676. doi: 10.3390/microorganisms7120676 (PMC6955753; doi:10.3390/microorganisms7120676)
Supplement: Supplementary file 1 [file microorganisms-07-00676-s001.zip › Supplementary files /Table S3.docx]

**Table S3.** The relative abundance at genus level of Proteobacteria (Top 42). CK, no biochar and no *R. solanacearum* inoculation; Rs, *R. solanacearum* inoculation without biochar amendment; BC, biochar addition without *R. solanacearum* inoculation; Rs+BC, biochar amendment and *R. solanacearum* inoculation.

|  | **Treatments** | | | | | | | | | | | |
| --- | --- | --- | --- | --- | --- | --- | --- | --- | --- | --- | --- | --- |
| **Geuns** | **CK** | | | **BC** | | | **Rs** | | | **Rs+bc** | | |
|  | **Mean** | **Error** | **Sig.** | **Mean** | **Error** | **Sig.** | **Mean** | **Error** | **Sig.** | **Mean** | **Error** | **Sig.** |
| *unclassified* | 0.0196 | 0.0008 | b | 0.0204 | 0.0014 | b | 0.0210 | 0.0005 | ab | 0.0242 | 0.0003 | a |
| *unclassified* | 0.0172 | 0.0020 | a | 0.0177 | 0.0016 | a | 0.0229 | 0.0019 | a | 0.0220 | 0.0007 | a |
| *Sphingomonas* | 0.0113 | 0.0003 | b | 0.0148 | 0.0019 | ab | 0.0090 | 0.0014 | b | 0.0209 | 0.0036 | a |
| *unclassified* | 0.0103 | 0.0003 | a | 0.0113 | 0.0016 | a | 0.0109 | 0.0010 | a | 0.0162 | 0.0020 | a |
| *unclassified* | 0.0089 | 0.0010 | a | 0.0085 | 0.0006 | a | 0.0102 | 0.0006 | a | 0.0098 | 0.0004 | a |
| *unclassified* | 0.0062 | 0.0002 | b | 0.0075 | 0.0004 | ab | 0.0091 | 0.0011 | ab | 0.0106 | 0.0013 | a |
| *unclassified* | 0.0062 | 0.0003 | b | 0.0063 | 0.0002 | b | 0.0083 | 0.0010 | ab | 0.0098 | 0.0003 | a |
| *unclassified* | 0.0060 | 0.0001 | a | 0.0067 | 0.0011 | a | 0.0052 | 0.0005 | a | 0.0093 | 0.0016 | a |
| *Ralstonia* | 0.0027 | 0.0005 | a | 0.0024 | 0.0001 | a | 0.0099 | 0.0064 | a | 0.0103 | 0.0019 | a |
| *Burkholderia* | 0.0052 | 0.0003 | a | 0.0056 | 0.0003 | a | 0.0067 | 0.0008 | a | 0.0069 | 0.0003 | a |
| *unclassified* | 0.0046 | 0.0003 | b | 0.0050 | 0.0003 | b | 0.0057 | 0.0003 | ab | 0.0070 | 0.0004 | a |
| *Thauera* | 0.0043 | 0.0004 | a | 0.0047 | 0.0005 | a | 0.0054 | 0.0005 | a | 0.0057 | 0.0002 | a |
| *unclassified* | 0.0038 | 0.0002 | b | 0.0067 | 0.0009 | a | 0.0044 | 0.0006 | ab | 0.0047 | 0.0002 | ab |
| *Geobacter* | 0.0025 | 0.0002 | b | 0.0056 | 0.0012 | a | 0.0031 | 0.0005 | ab | 0.0032 | 0.0001 | ab |
| *Thiobacillus* | 0.0027 | 0.0001 | b | 0.0025 | 0.0001 | b | 0.0032 | 0.0002 | ab | 0.0037 | 0.0002 | a |
| *unclassified* | 0.0029 | 0.0003 | a | 0.0024 | 0.0002 | a | 0.0035 | 0.0001 | a | 0.0033 | 0.0004 | a |
| *unclassified* | 0.0024 | 0.0001 | a | 0.0026 | 0.0002 | a | 0.0032 | 0.0008 | a | 0.0038 | 0.0006 | a |
| *Devosia* | 0.0021 | 0.0003 | b | 0.0026 | 0.0002 | ab | 0.0025 | 0.0006 | ab | 0.0043 | 0.0006 | a |
| *unclassified* | 0.0025 | 0.0003 | a | 0.0026 | 0.0003 | a | 0.0028 | 0.0002 | a | 0.0036 | 0.0001 | a |
| *unclassified* | 0.0022 | 0.0002 | b | 0.0027 | 0.0002 | ab | 0.0025 | 0.0001 | b | 0.0035 | 0.0003 | a |
| *Hyphomicrobium* | 0.0023 | 0.0003 | a | 0.0029 | 0.0006 | a | 0.0026 | 0.0003 | a | 0.0030 | 0.0002 | a |
| *Pseudomonas* | 0.0009 | 0.0002 | b | 0.0009 | 0.0001 | b | 0.0015 | 0.0004 | b | 0.0087 | 0.0022 | a |
| *Steroidobacter* | 0.0019 | 0.0002 | b | 0.0025 | 0.0003 | b | 0.0020 | 0.0003 | b | 0.0040 | 0.0001 | a |
| *unclassified* | 0.0022 | 0.0002 | a | 0.0024 | 0.0002 | a | 0.0024 | 0.0003 | a | 0.0027 | 0.0001 | a |
| *Anaeromyxobacter* | 0.0020 | 0.0001 | a | 0.0023 | 0.0002 | a | 0.0024 | 0.0002 | a | 0.0024 | 0.0001 | a |
| *Pseudoxanthomonas* | 0.0012 | 0.0004 | a | 0.0005 | 0.0000 | a | 0.0016 | 0.0004 | a | 0.0055 | 0.0026 | a |
| *Massilia* | 0.0021 | 0.0005 | a | 0.0013 | 0.0000 | a | 0.0026 | 0.0008 | a | 0.0028 | 0.0005 | a |
| *Luteimonas* | 0.0013 | 0.0001 | a | 0.0019 | 0.0000 | a | 0.0014 | 0.0006 | a | 0.0040 | 0.0011 | a |
| *Microvirga* | 0.0019 | 0.0001 | a | 0.0022 | 0.0002 | a | 0.0019 | 0.0005 | a | 0.0024 | 0.0001 | a |
| *unclassified* | 0.0017 | 0.0001 | a | 0.0015 | 0.0001 | a | 0.0023 | 0.0003 | a | 0.0023 | 0.0003 | a |
| *Bradyrhizobium* | 0.0017 | 0.0002 | a | 0.0023 | 0.0003 | a | 0.0018 | 0.0000 | a | 0.0020 | 0.0001 | a |
| *Phenylobacterium* | 0.0011 | 0.0002 | b | 0.0012 | 0.0001 | b | 0.0013 | 0.0002 | b | 0.0039 | 0.0012 | a |
| *Rhizobium* | 0.0015 | 0.0002 | a | 0.0013 | 0.0002 | a | 0.0014 | 0.0003 | a | 0.0028 | 0.0008 | a |
| *Desulfuromonas* | 0.0016 | 0.0002 | a | 0.0017 | 0.0000 | a | 0.0017 | 0.0001 | a | 0.0018 | 0.0001 | a |
| *Sphingobium* | 0.0013 | 0.0002 | a | 0.0015 | 0.0002 | a | 0.0013 | 0.0001 | a | 0.0027 | 0.0005 | a |
| *Desulfocapsa* | 0.0013 | 0.0001 | a | 0.0013 | 0.0002 | a | 0.0014 | 0.0001 | a | 0.0016 | 0.0001 | a |
| *unclassified* | 0.0012 | 0.0000 | a | 0.0012 | 0.0002 | a | 0.0013 | 0.0002 | a | 0.0014 | 0.0000 | a |
| *Corallococcus* | 0.0008 | 0.0001 | a | 0.0010 | 0.0002 | a | 0.0016 | 0.0007 | a | 0.0014 | 0.0002 | a |
| *Ensifer* | 0.0008 | 0.0000 | a | 0.0008 | 0.0002 | a | 0.0011 | 0.0005 | a | 0.0019 | 0.0004 | a |
| *Rhodanobacter* | 0.0011 | 0.0001 | ab | 0.0009 | 0.0001 | b | 0.0011 | 0.0000 | ab | 0.0013 | 0.0000 | a |
| *unclassified* | 0.0011 | 0.0002 | a | 0.0010 | 0.0001 | a | 0.0011 | 0.0001 | a | 0.0011 | 0.0001 | a |
| *Hydrogenophaga* | 0.0015 | 0.0005 | a | 0.0006 | 0.0000 | a | 0.0009 | 0.0001 | a | 0.0010 | 0.0001 | a |
|  |  |  |  |  |  |  |  |  |  |  |  |  |
