# Supplementary material for: Biochar Suppresses Bacterial Wilt of Tomato by Improving Soil Chemical Properties and Shifting Soil Microbial Community
Source: Microorganisms. 2019 Dec 10;7(12):676. doi: 10.3390/microorganisms7120676 (PMC6955753; doi:10.3390/microorganisms7120676)
Supplement: Supplementary file 1 [file microorganisms-07-00676-s001.zip › Supplementary files /Table S2.docx]

**Table S2.** Taxonomic distribution of OTUs (97% identity) at the Bacteria phylum level of the rhizosphere soil. CK, no biochar and no *R. solanacearum* inoculation; Rs, *R. solanacearum* inoculation without biochar amendment; BC, biochar addition without *R. solanacearum* inoculation; Rs+BC, biochar amendment and *R. solanacearum* inoculation.

| **Bacteria phylum** | **Relative abundance** | | | |
| --- | --- | --- | --- | --- |
|  | **CK** | **BC** | **Rs** | **BC+Rs** |
| Acidobacteria | 0.1501 | 0.1091 | 0.1357 | 0.0857 |
| Actinobacteria | 0.0528 | 0.0534 | 0.0518 | 0.0744 |
| Armatimonadetes | 0.0042 | 0.0028 | 0.0038 | 0.0028 |
| BRC1 | 0.0004 | 0.0005 | 0.0006 | 0.0003 |
| Bacteroidetes | 0.0407 | 0.0528 | 0.0434 | 0.0643 |
| Chlamydiae | 0.0001 | 0.0003 | 0.0001 | 0.0001 |
| Chlorobi | 0.0007 | 0.0008 | 0.0008 | 0.0008 |
| Chloroflexi | 0.0172 | 0.0184 | 0.0182 | 0.0145 |
| Deinococcus-Thermus | 0.0001 | 0.0001 | 0.0002 | 0.0002 |
| Firmicutes | 0.1496 | 0.1583 | 0.1415 | 0.1565 |
| Gemmatimonadetes | 0.0183 | 0.0237 | 0.0203 | 0.0275 |
| Nitrospira | 0.0039 | 0.0047 | 0.0060 | 0.0064 |
| OD1 | 0.0004 | 0.0002 | 0.0005 | 0.0004 |
| OP11 | 0.0001 | 0.0000 | 0.0001 | 0.0001 |
| Planctomycetes | 0.0329 | 0.0264 | 0.0326 | 0.0264 |
| Proteobacteria | 0.1799 | 0.1985 | 0.2148 | 0.2785 |
| SR1 | 0.0000 | 0.0000 | 0.0000 | 0.0000 |
| Spirochaetes | 0.0007 | 0.0003 | 0.0005 | 0.0006 |
| Synergistetes | 0.0001 | 0.0001 | 0.0001 | 0.0001 |
| TM7 | 0.0000 | 0.0000 | 0.0000 | 0.0000 |
| Tenericutes | 0.0000 | 0.0001 | 0.0000 | 0.0001 |
| Thermotogae | 0.0007 | 0.0002 | 0.0004 | 0.0003 |
| Verrucomicrobia | 0.0361 | 0.0263 | 0.0345 | 0.0270 |
| WS3 | 0.0002 | 0.0000 | 0.0002 | 0.0001 |
| unclassified | 0.1745 | 0.1524 | 0.1637 | 0.1421 |
