# Supplementary material for: Biochar Suppresses Bacterial Wilt of Tomato by Improving Soil Chemical Properties and Shifting Soil Microbial Community
Source: Microorganisms. 2019 Dec 10;7(12):676. doi: 10.3390/microorganisms7120676 (PMC6955753; doi:10.3390/microorganisms7120676)
Supplement: Supplementary file 1 [file microorganisms-07-00676-s001.zip › Supplementary files /Table S1.docx]

Table S1. Alpha diversity index of microbial communities in various soil samples. CK, no biochar and no R. solanacearum inoculation; Rs, R. solanacearum inoculation without biochar amendment; BC, biochar addition without R. solanacearum inoculation; Rs+BC, biochar amendment and R. solanacearum inoculation.

| **Samples** | **Number of sequences** | **Coverage** | **Shannon** | **Simpson** | **ACE** | **CHAO** |
| --- | --- | --- | --- | --- | --- | --- |
| CK1 | 25777 | 0.93727 | 6.706 | 0.993 | 5836 | 5435 |
| CK2 | 44404 | 0.959688 | 6.383 | 0.989 | 7522 | 5761 |
| CK2 | 39874 | 0.958419 | 6.455 | 0.988 | 7203 | 5575 |
| BC1 | 38915 | 0.915739 | 6.597 | 0.990 | 13655 | 9367 |
| BC2 | 51318 | 0.964184 | 6.473 | 0.990 | 7808 | 6001 |
| BC3 | 23106 | 0.940145 | 6.164 | 0.985 | 4953 | 4655 |
| Rs1 | 69545 | 0.972608 | 6.534 | 0.990 | 8374 | 6227 |
| Rs2 | 78017 | 0.974352 | 6.635 | 0.992 | 8770 | 6700 |
| Rs3 | 43036 | 0.957687 | 6.747 | 0.994 | 7966 | 6117 |
| Rs+BC1 | 77943 | 0.951298 | 6.749 | 0.993 | 12350 | 8572 |
| Rs+BC2 | 58166 | 0.968057 | 6.343 | 0.986 | 8030 | 6026 |
| Rs+BC3 | 93456 | 0.97922 | 6.736 | 0.995 | 8488 | 6398 |
